# Inhibition of MRGPRX2 but not FcεRI or MrgprB2-mediated mast cell degranulation by a small molecule inverse receptor agonist

**DOI:** 10.3389/fimmu.2022.1033794

**Published:** 2022-10-06

**Authors:** Maram Bawazir, Aetas Amponnawarat, Yvonne Hui, Carole A. Oskeritzian, Hydar Ali

**Affiliations:** ^1^ Department of Basic and Translational Sciences, School of Dental Medicine, University of Pennsylvania, Philadelphia, PA, United States; ^2^ Department of Oral Diagnostic Sciences, Faculty of Dentistry, King Abdulaziz University, Jeddah, Saudi Arabia; ^3^ Department of Family and Community Dentistry, Faculty of Dentistry, Chiang Mai University, Chiang Mai, Thailand; ^4^ Department of Pathology, Microbiology and Immunology, University of South Carolina School of Medicine, Columbia, SC, United States

**Keywords:** mast cell (MC), MRGPRX2, PAMP-12, substance P, rocuronium, antagonist, MRGPRX2 antagonist

## Abstract

Mas-related G protein-coupled receptor-X2 (MRGPRX2) expressed on mast cells (MCs) contributes to hypersensitivity reactions to cationic US-Food and Drug Administration (FDA) approved drugs such as the neuromuscular blocking agent, rocuronium. In addition, activation of MRGPRX2 by the neuropeptide substance P (SP) and the pro-adrenomedullin peptide (PAMP-12) is associated with a variety of cutaneous conditions such as neurogenic inflammation, pain, atopic dermatitis, urticaria, and itch. Thus, small molecules aimed at blocking MRGPRX2 constitute potential options for modulating IgE-independent MC-mediated disorders. Two inverse MRGPRX2 agonists, named C9 and C9-6, have recently been identified, which inhibit basal G protein activation and agonist-induced calcium mobilization in transfected HEK293 cells. Substance P serves as a balanced agonist for MRGPRX2 whereby it activates both G protein-mediated degranulation and β-arrestin-mediated receptor internalization. The purpose of this study was to determine if C9 blocks MRGPRX2’s G protein and β-arrestin-mediated signaling and to determine its specificity. We found that C9, but not its inactive analog C7, inhibited degranulation in RBL-2H3 cells stably expressing MRGPRX2 in response to SP, PAMP-12 and rocuronium with an IC_50_ value of ~300 nM. C9 also inhibited degranulation as measured by cell surface expression of CD63, CD107a and β-hexosaminidase release in LAD2 cells and human skin-derived MCs in response to SP but not the anaphylatoxin, C3a or FcϵRI-aggregation. Furthermore, C9 inhibited β-arrestin recruitment and MRGPRX2 internalization in response to SP and PAMP-12. We found that a G protein-coupling defective missense MRGPRX2 variant (V282M) displays constitutive activity for β-arrestin recruitment, and that this response was significantly inhibited by C9. Rocuronium, SP and PAMP-12 caused degranulation in mouse peritoneal MCs and these responses were abolished in the absence of MrgprB2 or cells treated with pertussis toxin but C9 had no effect. These findings suggest that C9 could provide an important framework for developing novel therapeutic approaches for the treatment of IgE-independent MC-mediated drug hypersensitivity and cutaneous disorders.

## Introduction

Mast cells (MCs) are tissue-resident immune cells that arise from hematopoietic lineage and are found close to peripheral nerve endings and blood vessels beneath the surface epithelium (1, 2). Although MCs are critical for host defense against microbial infection, they are best known for their roles in allergic disorders such as food allergy and asthma (1). These reactions are initiated following the cross-linking of high-affinity IgE receptors (FcϵRI) by allergens resulting in the release of histamine from secretory granules followed by newly synthesized mediators (3). Hypersensitivity reactions to Food and Drug Administration (FDA)-approved drugs such as neuromuscular blocking agents (NMBAs), fluoroquinolones, vancomycin, and radiographic contrast agents occur as a result of MC activation (1, 4). Since most patients with hypersensitivity reactions to NMBAs have not been previously exposed to the drug (5), an IgE-independent activation of MCs likely contributes to these reactions but the receptors involved remained a mystery until recently. In addition to FcεRI, a subtype of MCs found predominantly in connective tissues express Mas-related G protein-coupled receptor X2 (MRGPRX2; mouse counterpart MrgprB2). The seminal observation by McNeil et al. (6), that all cationic drugs including NMBAs that induce hypersensitivity reactions activate human and mouse MCs *via* MRGPRX2 and MrgprB2, respectively solved the mystery as to how these drugs could induce IgE-independent adverse reactions.

A unique feature of MRGPRX2 is that in addition to FDA-approved cationic drugs, it can be triggered by a wide range of endogenous agonists such as the neuropeptides substance P (SP), cortistatin, neuromedin, pro-adrenomedullin peptide (PAMP-12), eosinophil-derived major basic proteins and the host defense peptides (1, 7–10). Furthermore, MRGPRX2 has recently been implicated in the pathogenesis of mastocytosis (11), neurogenic inflammation (12), chronic urticaria (13), chronic prurigo (8), rosacea (14, 15), atopic dermatitis (16), and allergic contact dermatitis/itch (17). Thus, given the new and emerging role of MRGPRX2 on the pathogenesis of a variety of cutaneous conditions, the development of high-affinity receptor inhibitors may provide a novel approach for the modulation of these IgE-independent MC-mediated disorders (18).

Current strategies to modulate MRGPRX2 function involve direct inhibition of the receptor and its downstream signaling (10, 18, 19). It is noteworthy that although human MRGPRX2 and mouse MrgprB2 are activated by the same group of cationic ligands, there are important differences in the concentrations required to activate these receptors. Thus, EC_50_ value of SP towards MRGPRX2 is ~360-fold lower than that for MrgprB2. By contrast, the EC_50_ value of rocuronium for MRGPRX2 is ~12-fold higher than that for MrgprB2 (6, 20). This difference is attributed to the low amino acid sequence identity (~53%) between MRGPRX2 and MrgprB2 (21). Surprisingly, however, many of the small molecule inhibitors that attenuate MRGPRX2-mediated responses in human MCs *in vitro* also block MrgprB2-mediated responses *in vivo*. For example, it is generally accepted that the cathelicidin LL-37, which is elevated in the skin of rosacea patients, contributes to the disease through the activation of MCs *via* MRGPRX2 (14, 22). Callahan et al. (15), recently showed that osthole, a natural plant coumarin, inhibits LL-37-induced degranulation of human skin MCs *in vitro* and prevents LL-37-induced rosacea in mice. The authors proposed that osthole allosterically modulates MRGPRX2, and perhaps MrgprB2, to prevent LL-37-induced rosacea in mice. However, Roy et al. (14), showed that while MCs are required for LL-37-induced rosacea in mice, MrgprB2 does not fully mediate this response. In addition to MrpgrB2, mouse connective tissue MCs express transcripts for MrgprB1, MrgprB8, and MrgprB13 (23, 24). It is therefore possible that in addition to MrgprB2, one or more of these receptors contribute to rosacea-like inflammation in mice and that osthole prevents this response by modulating multiple receptors in mouse MCs. Thus, to develop specific small molecule inhibitors to modulate MRGPRX2-mediated disorders, it is first critical to identify compounds that selectively inhibit MRGPRX2 but not MrgprB2 or other receptor-mediated signaling and mediator release *in vitro.*

In 2019, Ogasawara et al. (19), of Japan Tobacco Inc. screened an in-house library consisting of ~12,000 commercially available compounds for their ability to inhibit SP-induced Ca^2+^ mobilization in HEK293 cells stably expressing MRGPRX2. The authors identified two compounds designated as compound 1 (MW 223.6) and compound 2 (MW 223.6) that inhibit MRGPRX2-mediated calcium (Ca^2+^) mobilization and MC degranulation with an IC_50_ value of ~2 µM. However, for therapeutic utility, it is desirable to develop compounds that specifically block MRGPRX2 at concentrations lower than those reported for compounds 1 and 2. Recently, Cao et al. (25), utilized compounds 1 and 2 as templates to search the ZINC database (http://zinc15.docking.org) and performed two rounds of analog modeling in the ultra-large make-on-demand library. This led to the identification of two compounds, named C9 (MW 275) and C9-6 (MW 258) that serve as inverse agonists to inhibit MRGPRX2-mediated G protein activation and Ca^2+^ mobilization in transfected HEK293 cells without blocking responses to a number of other GPCRs (25). However, the possibility that these compounds inhibit MC degranulation in response to SP, PAMP-12, and rocuronium has not been determined. Also, their effects on MrgprB2 and IgE-mediated responses have not been determined.

In addition to G proteins, many MRGPRX2 agonists also cause β-arrestin recruitment to promote receptor internalization (26, 27). Furthermore, many naturally occurring missense MRGPRX2 variants have been identified that may contribute to the receptor’s role in either protecting from or promoting MC-mediated disorders (1, 28, 29). In the present study, we first sought to determine the effects of C9 on MRGPRX2-mediated G protein activation, Ca^2+^ mobilization, degranulation, β-arrestin recruitment, and receptor internalization. We then asked whether it could modulate the constitutive activity of a missense variant of MRGPRX2. Finally, we sought to evaluate the specificity of C9 for MRGPRX2 by testing its ability to inhibit MrgprB2 and FcεRI-mediated degranulation in MCs. The data presented herein suggest that C9 could provide an important framework for developing novel and specific high-affinity small molecules to modulate MRGPRX2-mediated drug hypersensitivity and inflammatory disorders.

## Materials and methods

### Reagents

Cell culture reagents were purchased from Invitrogen (Carlsbad, CA); PAMP-12 from MedChem Express (Monmouth, NJ); Substance P from AnaSpec (Fremont, CA): Rocuronium from the Cayman chemical (Hayward, CA). Recombinant mouse interleukin-3 (IL-3), mouse stem cell factor (SCF), and recombinant human SCF (rhSCF) were obtained from Peprotech (Rocky Hill, NJ). Compounds C9 (2-sulfanyl-3-[(1,3-thiazol-2-yl)methyl]-3,4-dihydroquinazolin-4-one, MW 275), C9-6 (3-[furan-2-yl)methyl]-2-sulfanyl-3,4-dihydroquinazolin-4-one, MW. 258), and C7 (3-[2-(pyridin-2-yl)ethyl]-2-sulfanyl-3,4-dihydroquinazolin-4-one, MW 283), were obtained from Enamine LLC (Monmouth, NJ). P-nitrophenyl-N-acetyl-b-D-glucosamine (PNAG) and dimethyl sulfoxide (DMSO) were obtained from Sigma-Aldrich (St. Louis, MO). Fura-2 acetoxymethyl ester from Abcam (Cambridge, MA). Bright-Glo Luciferase was from Promega (Madison, WI). Lipofectamine™ 2000 transfection reagent from Invitrogen (Carlsbad, CA). PE anti-human MRGPRX2 antibody (Clone K125H4, Catalog 359004), FITC anti-human FcϵRIα (Clone AER-37, Catalog 334608), purified anti-human FcϵRIα (Clone AER-37, Catalog 334602), APC anti-human CD63 (Clone H5C6, Catalog 353008), and FITC anti-human CD107a (Clone H4A3, Catalog 328606) antibodies were from BioLegend (San Diego, CA). PE IgG2b Isotype control (Clone eB149/10H5, Catalog 12-4031-81) and FITC IgG Isotype control (Clone eBio299Arm, Catalog 11-4888-81) antibodies were from Invitrogen (Carlsbad, CA). Pertussis toxin (PTx) was obtained from List Biological Laboratories (Campbell, CA).

### Mice

Mice were housed in pathogen-free conditions and autoclaved hardwood bedding. Mice aged from 8 to 10 weeks old, and both genders were used in all experiments. C57BL/6 mice were obtained from the Jackson Laboratory (Bar Harbor, ME, USA), and mice MrgprB2^−/−^ mice were generated using CRISPR/Cas9 technology by CRISPR core of the University of Pennsylvania as previously described (30). Approval for the use of mice was obtained from the Institutional Animal Care and Use Committee at the University of Pennsylvania.

### Cell line culture

RBL-2H3 cells stably expressing human MRGPRX2 (RBL-MRGPRX2) were cultured in Dulbecco’s modified Eagle’s medium (DMEM) supplemented with 10% FBS, L-glutamine (2 mM), penicillin (100 IU/ml), streptomycin (100 mg/ml) and 1 mg/ml G418 (31). HTLA cells and cells stably expressing MRGPRX2-Tango (HTLA-MRGPRX2) were cultured similarly to RBL-2H3 cells in addition to the presence of hygromycin B (200 μg/ml), puromycin (5 mg/ml), and G418 (500 μg/ml) (32, 33). Laboratory of Allergic Diseases 2 (LAD2) cells were provided by Dr. A. Kirshenbaum and Dr. D. Metcalfe (National Institute of Allergy and Infectious Diseases, National Institutes of Health, Bethesda, MD, USA). Cells were maintained in a complete StemPro-34 medium supplemented with L-glutamine (2 mM), penicillin (100 IU/ml), streptomycin (100 μg/ml), and 100 ng/ml recombinant human stem cell factor (rhSCF). Weekly hemidepletions using media containing rhSCF were carried out as described (34).

### Mouse peritoneal mast cell isolation and culture

Cells were isolated from peritoneal lavages of WT and MrgprB2^−/−^ mice and were cultured for 4–8 weeks in complete Roswell Park Memorial Institute 1640 Medium (RPMI 1640) containing 10% FCS, penicillin (100 IU/ml), streptomycin (100 mg/ml), recombinant mouse IL-3 (10 ng/ml) and SCF (30 ng/ml). Cells were used within 4–8 weeks (27, 35). All cell cultures were kept at a 37°C incubator with 5% CO_2_. Suspension cells were used later in the experiments as peritoneal MCs (PMCs) and purity of the cultured cells was determined by flow cytometry using anti-mouse c-kit and FcϵRI antibodies (20).

### Human skin-derived mast cell isolation and culture

Surgical skin samples were obtained through the Cooperative Human Tissue Network of the National Cancer Institute, as approved by the University of South Carolina’s Internal Review Board. Human skin MCs were collected and cultured from three individual donors, as described previously (20, 36). Briefly, subcutaneous fat was removed by blunt dissection, and the remaining tissue was chopped into 1- to 2-mm pieces before being digested for 2 h at 37°C with type 2 collagenase (1.5 mg/ml), hyaluronidase (0.7 mg/ml), and type 1 DNase (0.3 mg/ml) in HBSS. The dispersed cells were collected and resuspended in HBSS containing 1% FCS and 10 mM HEPES and filtered through a No. 80 mesh sieve. Cells were then resuspended in HBSS and layered over 75% Percoll in an HBSS cushion and centrifuged at 800×g at 37°C for 20 min. The interface between the buffer and Percoll was used to collect the nucleated cells. Percoll gradient-enriched cells were resuspended in a serum-free X-VIVO 15 medium containing rhSCF (100 ng/ml) at a concentration of 1 × 10^6^ cells/ml. MCs were used after 6–10 weeks of culture, toluidine blue staining was utilized to confirm the purity of MCs was approximately 100%.

### Degranulation measured by β-hexosaminidase release assay

RBL-MRGPRX2 cells (5×10^4^ cells/well), LAD2 cells (1×10^4^ cells/well), primary human skin-derived MCs (5×10^3^ cells/well), and PMCs (1×10^4^ cells/well) were seeded into a 96-well plate in a total volume of 50 μl HEPES buffer containing 0.1% bovine serum albumin (BSA). Cells were preincubated in the absence or presence of MRGPRX2 antagonist for 5 min followed by the addition of SP, PAMP-12, or rocuronium for 30 min at 37°C. To determine the total β-hexosaminidase, cells were lysed in 50 μl of 0.1% Triton X-100. Aliquots (20 μl) of supernatants were incubated with 20 μl of 1 mM p-nitrophenyl-N-acetyl-b-D-glucosamine (PNAG) at 37°C for 1 h (RBL-MRGPRX2 cells), and 1.5 h (LAD2, primary human skin-derived MCs, and PMCs). Finally, 250 μl of stop solution was added (0.1 M Na_2_CO_3_/0.1 M NaHCO_3_) to stop the reaction. The absorbance was measured with a microplate reader at a wavelength of 405 nm using a Versamax microplate spectrophotometer (Molecular Devices, San Jose, CA). Percentage of β-hexosaminidase degranulation was calculated by dividing the β-hexosaminidase release in the sample by total β-hexosaminidase release (37).

### Degranulation measured by the surface expression of CD107a and CD63

MC degranulation was also assessed by flow cytometric measurement of cell surface CD107a (Lysosomal-Associated Membrane Protein 1, LAMP-1) and CD63 (LAMP-3) following agonist stimulation (38–40). LAD2 cells and human skin-derived MCs (1×10^5^ cells) were pre-incubated with compounds C9, C7, or vehicle control for 5 min at 37°C then stimulated with SP (0.3 μM), anti-human FcϵRIα (Clone AER-37; 0.3 μg/ml) (41) or C3a (3 nM) for 5 min at 37°C. The cells were then immediately fixed with fixation buffer (Biolegend, Catalog 420801) for 15 min at room temperature. After washing with FACS buffer (PBS containing 2% fetal calf serum [FCS] and 0.02% sodium azide), non-specific binding was blocked with 1% BSA in PBS for 30 min at 4°C. Cells were exposed to FITC-conjugated anti-CD107a and APC-conjugated anti-CD63 antibodies for 30 min at 4°C. Cell surface expression of CD107a and CD63 were analyzed by a BD LSR II flow cytometer (San Jose, CA) and FlowJo software version 10.7.2 (Tree Star Inc., Ashland, OR).

### Calcium mobilization assay

RBL-MRGPRX2 cells (2 × 10^6^ cells) were loaded with Fura-2 acetoxymethyl ester (1 μM) in HEPES buffer containing 0.1% BSA for 30 min in the dark at 37°C, followed by 15 min period for de-esterification in dark at room temperature. Cells were washed and resuspended in HEPES-buffered saline and Ca^2+^ mobilization was measured for the designated time. Calcium mobilization was determined by measuring the fluorescence ratio between dual excitation wavelengths of 340 and 380 nm, and an emission wavelength of 510 nm using a Hitachi F-2700 Fluorescence Spectrophotometer (29).

### Generation of HTLA cells transiently expressing MRGPRX2 and V282M missense

Transient transfection of HTLA cells to express MRGPRX2 or its missense V282M variant was performed as described previously (29, 35). Briefly, HTLA cells (2×10^6^ cells) were plated in a 6-well plate in a complete medium and incubated overnight at 37°C. Cells were transiently transfected the next day with 2 μg of MRGPRX2 or its variant V282M in Tango plasmids using Lipofectamine™ 2000 DNA transfection reagent, according to the manufacturer protocol. Cells were then incubated in an antibiotic-free medium (DMEM supplemented with 10% FBS and L-glutamine) overnight at 37°C with 5% CO_2_ and used within 16-24 h after transfection.

### Transcriptional activation following arrestin translocation (TANGO) assay

HTLA cells stably expressing MRGPRX2 (5×10^4^ cells/well) were plated into a 96-well plate with an antibiotic-free medium and incubated for 6 h to allow cells’ adherence at 37^°^C with 5% CO_2_. Following 6 h of incubation, the medium was aspirated, and cells were incubated with C9 (1 or 10 μM) in an antibiotic-free medium for 5 min, followed by stimulation with MRGPRX2 agonist for an additional 16 h at 37^°^C with a 5% CO_2_ incubator. After 16 h, the medium was aspirated and replaced with 100 μl of Bright-Glo solution (Promega). The relative luminescence unit was measured in a Thermo Labsystems Luminoskan Ascent 392 Microplate Luminometer (42). For HTLA cells transiently transfected with MRGPRX2 or its missense variant, V282M tango plasmid, cells treated with C9 (10 μM), C7 (10 μM), or non-treated control and assay were performed similarly (32).

### MRGPRX2 internalization

RBL-MRGPRX2 or HTLA-MRGPRX2 cells (0.5x10^6^ cells), and LAD2 cells (0.3x10^6^ cells) were stimulated with SP, PAMP-12, and rocuronium in the presence and absence of C9. Cells were washed and suspended in FACS buffer (PBS containing 2% fetal calf serum [FCS] and 0.02% sodium azide) and incubated with the PE-conjugated anti-MRGPRX2 antibody for 30 min at 4°C in the dark. Cells were washed in FACS buffer, fixed in 1.5% paraformaldehyde and acquired using a BD LSR II flow cytometer (San Jose, CA) and analyzed with the FlowJo software version 10.8.1 (Tree Star Inc., Ashland, OR).

### Statistical analysis

GraphPad PRISM software version 9.0.1 (San Diego, CSA) was used to perform the statistical analysis. Results were expressed as mean ± standard error of the mean (SEM) values. SEM values were derived from three independent experiments. Statistical significance was measured by t-test for single comparisons and one-way analysis of variance (ANOVA) or two-way ANOVA for multiple comparisons. A *P*-value ≤ 0.05 was considered to be significant.

## Results

### Compounds C9 and C9-6 inhibit substance P, PAMP-12, and rocuronium-induced degranulation and Ca^2+^ mobilization in RBL-2H3 cells stably expressing MRGPRX2

**Figure 1:**
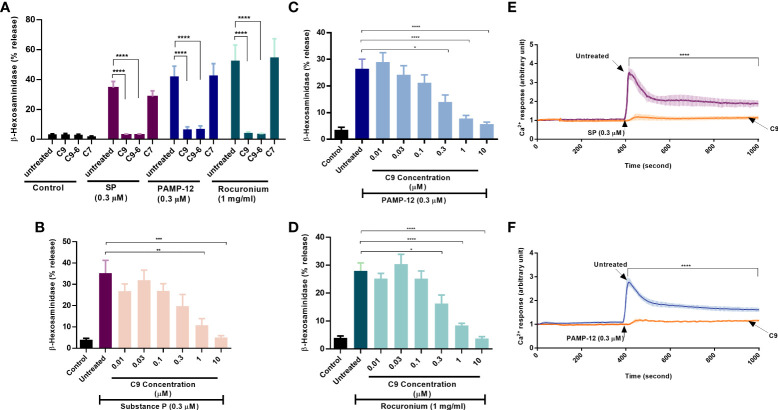
Compounds C9 and C9-6 inhibit substance P, PAMP-12, and rocuronium-induced degranulation and Ca^2+^ mobilization in RBL-2H3 cells stably expressing MRGPRX2. **(A)** RBL-MRGPRX2 cells were preincubated with C9, C9-6, or C7 (10 µM, 5 min) before exposure to indicated concentrations of agonists for 30 min, and the degranulation was determined by quantitating β-hexosaminidase release. **(B, C, D)** RBL-MRGPRX2 cells were preincubated with different concentrations of C9 (5 min) followed by agonist stimulation and β-hexosaminidase release was measured. **(E,F)** Cells were preincubated with C9 (DMSO for control) and Ca^2+^ mobilization was determined following stimulation with SP or PAMP-12 (0.3 µM, 10 min). Data are the mean ± SEM of at least three experiments. Statistical significance was determined using one-way ANOVA test at a value *P < 0.05, **P < 0.01, ***P < 0.001 and ****P < 0.0001.

ZINC-3573 has been identified as a potent MRGPRX2 agonist (33). Using calcium mobilization as an assay in HEK293 cells stably expressing MRGPRX2, Cao et al. (25), showed that C9 and C9-6 inhibit ZINC-3573-induced responses with *K*i values of 43 nM and 58 nM, respectively. C9 also inhibited ZINC3573-induced degranulation in a human MC line (LAD2 cells) endogenously expressing MRGPRX2 but with an IC_50_ value of >1 µM (25). However, an inactive analog of C9, known as C7, had no inhibitory activity on ZINC3573-induced responses in HEK293 cells or LAD2 cells. We first sought to determine the effects of C9, C9-6, and C7 on degranulation in response to two endogenously generated MRGPRX2 agonists (SP and PAMP-12) that contribute to a variety of MC-dependent disorders and an FDA-approved drug that is associated with hypersensitivity (rocuronium). For this, we preincubated RBL-2H3 cells stably expressing MRGPRX2 (RBL-MRGPRX2) with C9, C9-6, or C7 (10 µM, 5 min) and tested their effects on β-hexosaminidase release in response to SP (0.3 µM), PAMP-12 (0.3 µM) and rocuronium (1 mg/ml). As shown in **Figure 1A**, C9 and C9-6 abolished degranulation in response to these agonists but C7 had no effect. For subsequent studies, we used C9 and the inactive analog C7, where appropriate.

To determine the minimal concentration of C9 required to cause significant inhibition of degranulation, we preincubated RBL-MRGPRX2 cells with different concentrations of C9 (0.01 µM – 10 µM, 5 min) and assessed β-hexosaminidase release in response to SP, PAMP-12, and rocuronium. C9 inhibited degranulation in response to all agonists tested in a dose-dependent manner with an IC_50_ value of ~0.3 µM **(** **Figures 1B–D** **)**. Because Ca^2+^ mobilization is required for MC degranulation, we tested the effect of C9 on SP and PAMP-12-induced Ca^2+^ response in RBL-MRGPRX2 cells. For this, Fura-2-loaded cells were preincubated with C9 (10 µM, 5 min) and exposed to SP or PAMP-12 (0.3 µM) and Ca^2+^ mobilization was measured continuously for the next 10 min. We found that C9 caused substantial inhibition of the Ca^2+^ response to both agonists at all time points tested (**Figures 1E, F**).

### C9 inhibits MRGPRX2-mediated degranulation in LAD2 cells and human skin MCs without affecting C3a receptor and FcεRI-mediated responses

**Figure 2:**
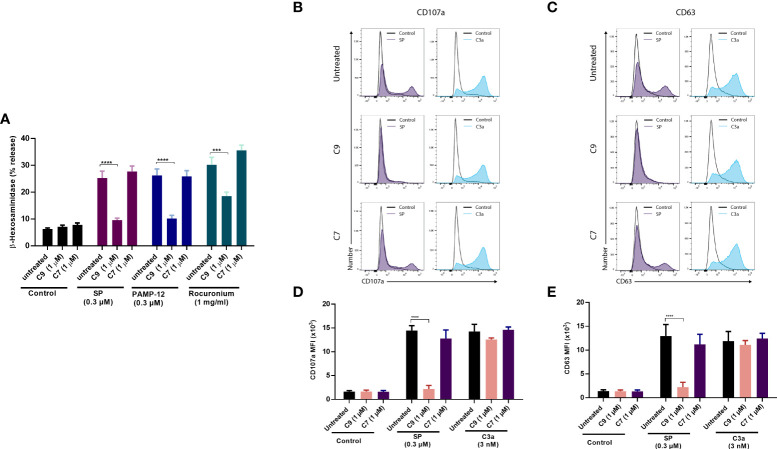
C9 inhibits MRGPRX2-mediated MC degranulation in LAD2 cells without affecting the C3aR response. **(A)** LAD2 cells were preincubated with C9 and inactive analog C7 (1 µM, for 5 min) then exposed to agonists at the indicated concentrations, and degranulation was assayed by measuring the release of β-hexosaminidase. **(B, C)** Cells were stimulated with SP (0.3 µM) or C3a (3 nM) for 5 min in the presence or absence of C9 or C7. Cell surface expression of CD107a and CD63 were determined by flow cytometry. Representative histograms of three independent experiments are shown. **(D, E)** The mean fluorescent intensity (MFI) levels of CD107a and CD63 are shown. Data are expressed as mean ± SEM. Statistical significance was determined by two-way ANOVA test at a value ***P < 0.001 and ****P < 0.0001.

LAD2 cell is a human MC line that endogenously expresses MRGPRX2 and C3a receptor (C3aR) and has been used extensively to GPCR function in MCs (35, 43, 44). We, therefore, sought to determine if C9 inhibits degranulation in response to MRGPRX2 agonists. As shown in **Figure 2A**, preincubation of LAD2 cells with C9, but not C7 (1 µM, 5 min), resulted in a substantial reduction of SP, PAMP-12, and rocuronium-induced degranulation as measured by β-hexosaminidase release. CD107a and CD63 are granule-associated proteins that undergo externalization during the degranulation (45). We found that both SP and the anaphylatoxin C3a, caused increased cell surface expression of CD107a and CD63, as measured by flow cytometry **(** **Figures 2B, C** **)**. Furthermore, preincubation of LAD2 cells with C9 (1 µM, 5 min), but not C7, abolished the SP response. By contrast, C9 or C7 had no effect on C3a-induced cell surface expression of CD107a and CD63. Quantitative results are shown in **Figures 2D, E**.

**Figure 3:**
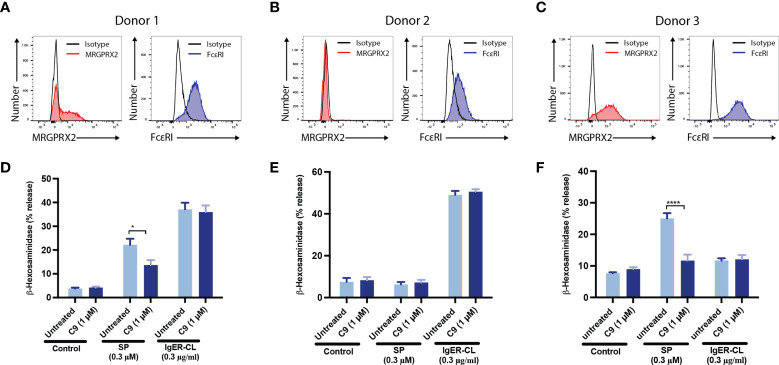
C9 inhibits MRGPRX2-mediated degranulation in human skin-derived MCs without affecting the FcεRI response. **(A-C)** Flow cytometry histograms demonstrating MRGPRX2 and FcεRI expression of cultured human skin-derived MCs from 3 healthy donors are shown. **(D-F)** Cells were preincubated with C9 (1 µM, 5 min) and exposed to SP (0.3 µM) or FcϵRIα-aggregation antibody, IgER-CL (IgE receptor cross-linking) (0.3 μg/ml) and β-hexosaminidase release was determined. Data are expressed as mean ± SEM. Statistical significance was determined by two-way ANOVA test at a value *P < 0.05, and ****P < 0.0001.

**Figure 4:**
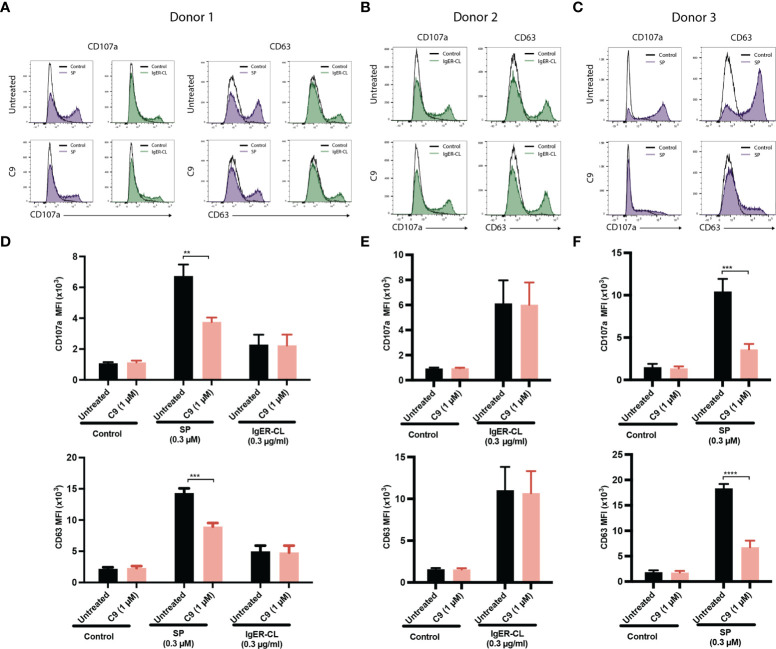
C9 inhibits the upregulation of cell surface expression of CD107a and CD63 in response to SP in human skin-derived MCs. **(A-C)** Cells from three donors were preincubated with buffer or C9 (1 µM, 5 min), stimulated with SP (0.3 µM) or FcϵRIα-aggregation antibody, IgER-CL (0.3 µg/ml) for an additional 5 min and cell surface expression of CD107a and CD63 was determined by flow cytometry. Representative histograms of CD107a and CD63 expression are shown. **(D–F)** The mean fluorescent intensity (MFI) levels of CD107a and CD63 are shown. Data are expressed as mean ± SEM. Statistical significance was determined by two-way ANOVA test at a value **P < 0.01, ***P < 0.001, and ****P < 0.0001.

To determine the biological relevance of the findings in LAD2 cells, we performed selected experiments with primary human skin-derived MCs obtained from three healthy donors. When the purity of cultured MCs reached ~100%, flow cytometry was used to determine MRGPRX2 and FcϵRI expression (**Figures 3A–C**). Consistent with MRGPRX2 expression, skin MCs from donors 1 and 3 responded to SP for degranulation, as assessed by β-hexosaminidase release and this response was significantly inhibited by C9 (1 µM) (**Figures 3D–F** **)**. Although MCs from all three donors expressed cell surface FcεRI, those from donors 1 and 2 responded to FcϵRIα-aggregation for β-hexosaminidase release but this response was resistant to inhibition by C9 (**Figures 3D–F** **)**. To further validate the specificity of C9, we tested its ability to inhibit cell surface CD107a and CD63 expression in response to SP and FcϵRIα-aggregation. In agreement with the β-hexosaminidase release, SP (donor 1 and donor 3) and FcϵRIα-aggregation (donor 1 and donor 2) induced significant upregulation of cell surface CD107a and CD63 (**Figures 4A–C**). C9 significantly blocked SP, but not FcϵRIα-aggregation responses (**Figures 4A–C**). The quantification of flow cytometry data is shown in **Figures 4D–F**. Together, these findings demonstrate that C9 specifically inhibits MRGPRX2-mediated MC degranulation without affecting the responses to C3a or FcϵRIα-aggregation.

### Pre-incubation of RBL-MRGPRX2 cells with C9 is not required for its ability to inhibit MRGPRX2-mediated Ca^2+^ mobilization and degranulation

**Figure 5:**
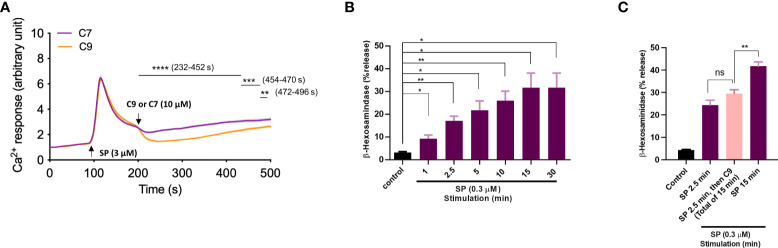
Pre-incubation of cells with C9 is not required for its ability to inhibit MRGPRX2-mediated Ca^2+^ mobilization and degranulation. **(A)** Ca^2+^ mobilization measurement of Fura-2 loaded RBL-MRGPRX2 cells following stimulation with SP (3 µM). As the response approached the plateau phase, the C9 or C7 (10 µM) was introduced and the response was measured for an additional 5 min. **(B)** RBL-MRGPRX2 cells were exposed to SP (0.3 µM) and the time-course of degranulation was assayed by measuring the release of β-hexosaminidase. **(C)** Cells were exposed to SP (0.3 µM) and degranulation was quantitated after 2.5 min (left, purple) or 15 min (right purple). Alternatively, cells were stimulated with SP for 2.5 min and then exposed to C9 (10 µM) for an extra 12.5 min (total of 15 min), and β-hexosaminidase release was determined (middle, pink). Data are expressed as mean ± SEM. Statistical significance was determined by unpaired *t*-test for single comparison and one-way ANOVA for multiple comparisons at a value *P < 0.05, **P < 0.01, ***P < 0.001, ****P < 0.0001, and ns denotes “not significant”.

For experiments described above, cells were preincubated with C9 for 5 min before exposure to stimulants. We next sought to determine if C9 could reverse an ongoing MRGPRX2-mediated response using Ca^2+^ mobilization as an assay in RBL-MRGPRX2 cells. As shown in **Figure 5A**, when C9 was added 100 sec after stimulation with SP, it caused an immediate and significant reduction of the response for the entire duration of the experiment when compared to C7 control. Our next goal was to determine the effect of C9 on ongoing degranulation in response to SP. For this, we first assessed the time-course (1 to 30 min) of SP-induced β-hexosaminidase release. As shown in **Figure 5B**, SP caused half-maximal degranulation at 2.5 min after stimulation and the reaction reached a maximal value at 15 min after stimulation. Next, for one set of experiments, we stopped the reaction at 2.5 min after stimulation with SP. For the second set, we added C9 at 2.5 min after SP stimulation and allowed degranulation to proceed until 15 min. As shown in **Figure 5C**, there was no significant difference in SP-induced degranulation at 2.5 min without C9 when compared to the response at 15 min after the addition of C9 at 2.5 min. These findings clearly demonstrate that C9 is able to stop an ongoing Ca^2+^ mobilization and degranulation in response to SP.

### C9 inhibits MRGPRX2-mediated β-arrestin recruitment and receptor internalization

**Figure 6:**
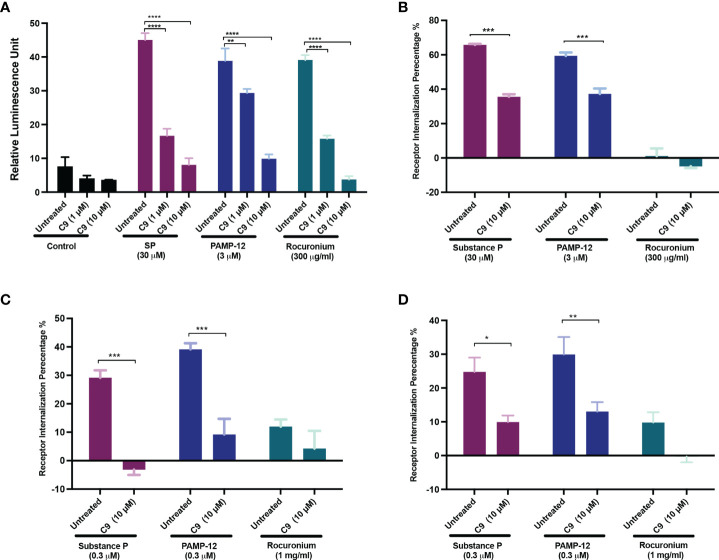
C9 inhibits MRGPRX2-mediated β-arrestin recruitment and receptor internalization. **(A)** HTLA-MRGPRX2 cells were preincubated with C9 (1 µM and 10 µM, 5 min) and then exposed to SP, PAMP-12, or rocuronium for 16h The medium was removed and substituted with the Bright-Glo solution (100 μl/well) in a 96-well plate. β-arrestin translocation-mediated gene expression was quantitated as a relative luminescence unit. **(B-D)** Quantitative analysis of receptor internalization in **(B)** HTLA-MRGPRX2, **(C)** RBL-MRGPRX2, and **(D)** LAD2 cells. Cells were preincubated with C9 (10 µM, 5 min), exposed to buffer or MRGPRX2 agonists (30 min) and cell surface receptor expression was determined by flow cytometry. The percentage of receptor internalization was calculated using a mean fluorescent intensity (MFI) in comparison to the untreated or vehicle controls. Data are expressed as mean ± SEM. Statistical significance was determined by two-way ANOVA test at a value *P < 0.05, **P < 0.01, ***P < 0.001, and ****P < 0.0001.

Using transcriptional activation following arrestin translocation (TANGO) assay in HTLA cells stably expressing MRGPRX2 (HTLA-MRGPRX2), we previously showed that SP causes β-arrestin translocation and that this is associated with receptor internalization (27, 33). However, the possibility that PAMP-12 causes β-arrestin signaling has not been determined. Thus, we first tested the ability of all 3 agonists to cause β-arrestin translocation using the TANGO assay. Cells were exposed to either buffer (control) or agonists for 16 h and β-arrestin-mediated gene expression (indicative of β-arrestin recruitment) was measured. As per previous report, we found that SP induced substantial β-arrestin recruitment, and that PAMP-12 also induced a similar response (**Figure 6A**). Lansu et al. (33), reported that rocuronium at a concentration of 100 µM does not induce TANGO in HTLA-MRGPRX2 cells. However, we found that rocuronium at a concentration of 300 µM induces a significant β-arrestin signal, which was similar in magnitude to that induced by SP and PAMP-12 (**Figure 6A**). To determine if C9 inhibits β-arrestin recruitment, we preincubated HTLA-MRGPRX2 cells with different concentrations of the antagonist and quantitated using the TANGO assay. We found that C9 at a concentration of 1 µM caused significant inhibition of response to all three agonists (**Figure 6A**).

To determine the effect of C9 on receptor internalization, HTLA-MRGPRX2 cells, RBL-MRGPRX2 cells, and LAD2 cells were preincubated with C9, then exposed to SP, PAMP-12, and rocuronium, and receptor internalization was assessed by determining loss of cell surface receptor expression by flow cytometry. In all three cell types, SP and PAMP-12 caused MRGPRX2 internalization and C9 inhibited this response but the magnitude of inhibition was greater in RBL cells when compared to HTLA, or LAD2 cells (**Figures 6B–D**). Interestingly, although rocuronium induced substantial β-arrestin recruitment, this was not associated with MRGPRX2 internalization in HTLA, RBL, or LAD2 cells (**Figures 6A–D**).

### Constitutive β-arrestin recruitment in response to a naturally occurring missense MRGPRX2 variant is inhibited by C9

**Figure 7:**
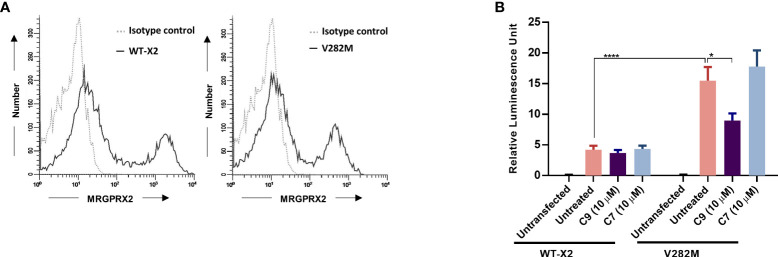
Constitutive β-arrestin recruitment in response to a naturally occurring missense MRGPRX2 variant is inhibited by C9. **(A)** HTLA cells transiently expressing MRGPRX2 (WT-X2) and its variant V282M were incubated with PE-conjugated anti-MRGPRX2 antibody or isotype controls and the cell surface receptor expression was determined by flow cytometry. **(B)** Cells were exposed to C9 (10 µM) or C7 (10 µM) without any agonist stimulation. After 16 h, the medium was removed and substituted with Bright-Glo solution (100 μl/well) in a 96-well plate. β-arrestin gene translocation-mediated gene expression was measured in a relative luminescence unit. Data are expressed as mean ± SEM. Statistical significance was determined by two-way ANOVA test at a value *P < 0.05 and ****P < 0.0001.

We recently identified a naturally occurring MRGPRX2 missense variant (V282M) that displays a loss of function phenotype for G protein-mediated degranulation (29, 35). We found that transient transfection of HTLA cells resulted in a similar level of cell surface expression of the V282M variant when compared to the wild-type receptor **(** **Figure 7A** **)**. Surprisingly, however, expression of V282M is associated with constitutive recruitment of β-arrestin in the absence of agonist stimulation. Interestingly, this response was significantly inhibited by C9 but not by its inactive analog, C7 **(** **Figure 7B** **)**.

### C9 does not inhibit MrgprB2-mediated degranulation in mouse peritoneal MCs

**Figure 8:**
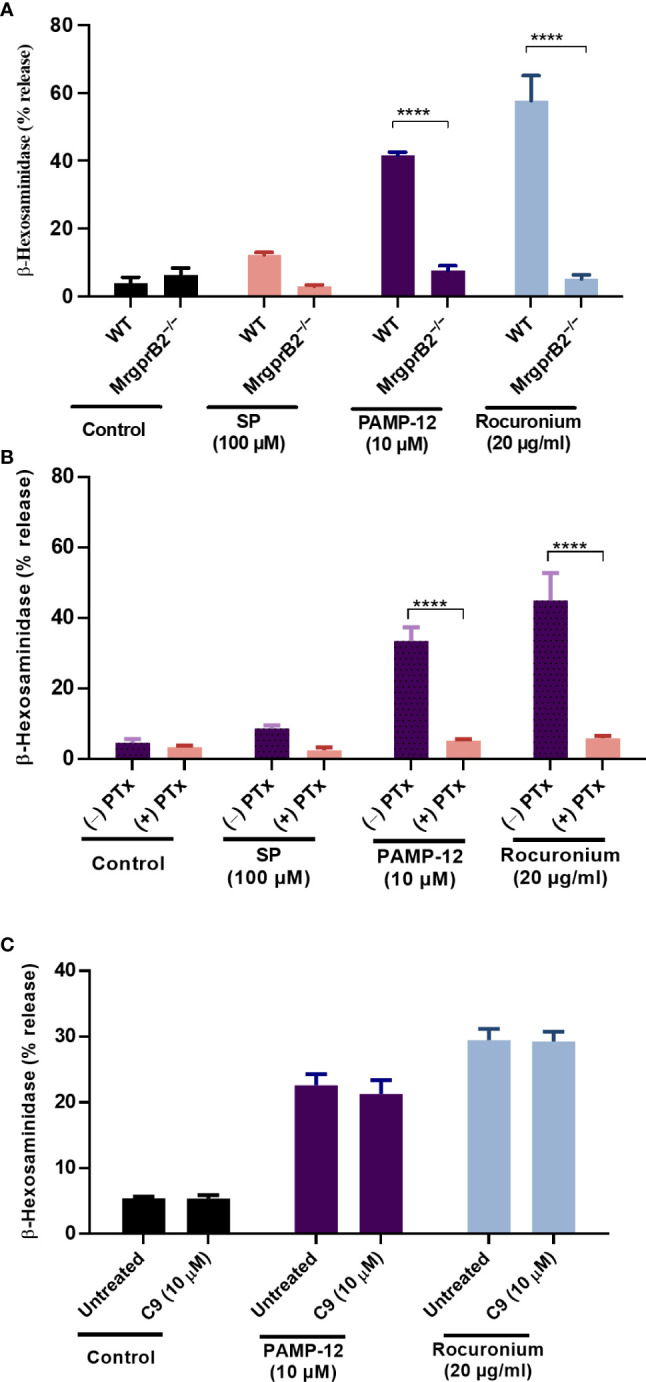
C9 does not inhibit MrgprB2-mediated degranulation in mouse peritoneal MCs. **(A)** Wild type (WT) and MrgprB2^−/−^ peritoneal-derived MCs (PMCs) were exposed to SP (100 µM), PAMP-12 (10 µM), and rocuronium (20 µg/ml) for 30 min and β-hexosaminidase release was measured. **(B)** WT-PMCs were cultured in the absence or presence of PTx (100 ng/mL, 16 h), exposed to SP, PAMP-12, or rocuronium for 30 min, and β-hexosaminidase release was determined. **(C)** WT-PMCs were preincubated with C9 (10 µM, 5 min), stimulated with PAMP-12 or rocuronium and β-hexosaminidase release was measured. All data points are the mean ± SEM of at least three experiments performed in triplicate. Statistical significance was determined by two-way ANOVA test at a value ****P < 0.0001.

Although MRGPRX2 and MrgprB2 share the same agonists there are important differences in the concentrations of SP and rocuronium required to activate these receptors (6). Consistent with previous reports, we found that SP at 100 µM induced only a small and variable degranulation, as measured by β-hexosaminidase release, but rocuronium at 20 µg/ml caused a substantial response in mouse PMCs **(** **Figure 8A** **)** (20). We first validated that degranulation in response to SP, PAMP-12, and rocuronium is mediated *via* MrgprB2 by demonstrating that PMCs cultured from MrgprB2^−/−^ mice do not respond to these agonists **(** **Figure 8A** **)**. Although inhibition of Gαi proteins by pertussis toxin (PTx) leads to almost complete inhibition of MRGPRX2-mediated degranulation in human CD34^+^ cell-derived MCs (19), its effect on MrgprB2-mediated degranulation is unknown. We therefore preincubated PMCs from WT mice with pertussis toxin (PTx; 100 ng/ml for 16 h) and tested the ability of SP, PAMP-12, and rocuronium to induce degranulation. The result presented in **Figure 8B** shows that PTx-pretreatment almost completely blocked β-hexosaminidase release in response to all three agonists, suggesting that, similar to MRGPRX2, MrgprB2-mediated response is mediated by Gαi. Next, we tested the ability of C9 (10 µM) to inhibit MrgprB2-mediated degranulation in PMCs generated from WT mice. Because SP induces a small and variable response, we utilized PAMP-12 and rocuronium for this set of experiments. Interestingly, we found that C9 was unable to inhibit MrgprB2-mediated β-hexosaminidase release (**Figure 8C**). The results from these studies demonstrate that similar to MRGPRX2 in human MCs, PAMP-12 and rocuronium couple to MrgprB2 and Gαi proteins but C9 is highly specific to human receptor MRGPRX2.

## Discussion

It is well documented that IgE-mediated MC activation participates in food allergy, anaphylaxis, and asthma. However, there has been an explosion in interest in the role of non-IgE-mediated MC activation, in particular MRGPRX2, on drug-induced hypersensitivity and a variety of cutaneous disorders. Cao et al. (25), recently identified C9, as a small molecule inverse agonist of MRGPRX2, which inhibits basal G protein activation and blocks ZINC-3573-induced calcium mobilization in transfected HEK293 cells. In the present study, we utilized transfected cell lines (RBL and HTLA), a human MC line endogenously expressing MRGRPX2 (LAD2), primary human skin-derived MCs, and mouse peritoneal MCs derived from WT and MrgprB2^−/−^ mice to show that C9 specifically inhibits MRGPRX2-mediated degranulation without affecting responses to FcεRI or MrgprB2.

Cao et al. (25), showed that C9 inhibits ZINC-3573-induced Ca^2+^ mobilization in transfected HEK293 cells with a Ki value 43 nM. However, C9 at 1 µM inhibited ZINC-3573-induced degranulation in LAD2 cells by <50% and required >10 µM for almost complete inhibition of degranulation. The reason for this difference is not clear but could reflect the agonist and cell types used. Our studies focused on three MRGPRX2 agonists that are implicated in a variety of cutaneous conditions and drug-induced hypersensitivity. However, unlike the situation with ZINC-3573 (25), C9 inhibited degranulation in response to SP, PAMP-12 and rocuronium with an IC_50_ value of ~300 nM and 1 µM of the compound caused substantial inhibition of the response. Cryo-EM structure of MRGPRX2 with a number of agonists has recently been resolved (25, 46). MRGPRX2 has two binding pockets, one with the negative charge and the other with hydrophobic residues (25). The neuropeptide cortistatin utilizes both pockets for binding whereas the synthetic small molecule MRGPRX2 agonist ZINC-3573 binds only to one pocket. Whether this difference reflects the high concentration of C9 required to inhibit degranulation induced by ZINC-3573 in LAD2 cells remains to be determined (25).

Since the description of CD63 as a surface degranulation marker, a flow cytometry-based assay known as basophil activation test (BAT) was developed and has been reliably used for the diagnosis of IgE-mediated allergic responses (47). As LAD2 cells and human peripheral CD34^+^ cell-derived MCs express MRGPRX2, a flow cytometric assay, known as MC activation test (MAT) that quantitates cell surface expression of CD63 and CD107a has been developed, which could be used to test MRGPRX2 activation by sera of patients undergoing drug hypersensitivity reactions (45, 48). Navines-Ferrer et al. (49), recently utilized CD63 upregulation as a measure of MC degranulation by sera obtained from healthy controls and patients who had experienced an anaphylactoid reaction due to drug administration. It was demonstrated that sera from patients who had undergone anaphylactoid reactions induced an upregulation of CD63 expression on LAD2 cells and that this response was reduced in MRGPRX2-silenced cells. Based on these findings, it was concluded that MRGPRX2 is a candidate for mediating hypersensitivity reactions. In the present study, we utilized MAT to show that while SP and C3a caused MC degranulation, C9 at 1 µM selectively blocked SP-induced response. In primary human skin-derived MCs, both SP and FcεRI-aggregation caused increased expression of CD63 and CD107a but C9 blocked the response to SP, but not FcεRI-aggregation. These findings suggest that C9 could be utilized as a novel diagnostic tool to determine the role of MRGPRX2 not only in drug-induced hypersensitivity but also in other MC-mediated disorders.

Sugammadex is a modified γ-cyclodextrin that reverses neuromuscular blockade by encapsulating rocuronium and atracurium and removing it from the neuromuscular junction. Coincubation of atracurium with sugammadex, in molar excess, inhibits atracurium and rocuronium-induced MRGPRX2-mediated MC activation (50, 51). However, given that sugammadex does not stop atracurium-induced MC degranulation, once initiated, it is unlikely to be useful in patients with ongoing atracurium or rocuronium-mediated perioperative anaphylaxis (51–53). We have shown that preincubation of RBL-MRGPRX2 cells with C9 is not required for its ability to block SP-induced Ca^2+^ mobilization and degranulation. Thus, when cells were exposed to SP for Ca^2+^ mobilization, the response was reversed almost immediately after C9 was added. Similarly, ongoing degranulation was also halted immediately after the addition of C9. These findings have clinical implications for the potential utilization of C9 or its derivatives for targeting MRGPRX2-mediated drug hypersensitivity and inflammatory disorders. Thus, it will be interesting to see if prophylactic administration of C9 or a similar drug before surgical procedures lowers overall perioperative patient mortality. Given that SP and PAMP-12 are implicated in urticaria, atopic dermatitis, and allergic contact dermatitis, it should be possible to utilize C9 or small molecules based on this compound for topical application to treat these conditions.

In addition to G protein, SP also couples to β-arrestin-mediated signaling to promote receptor internalization (27). We found that PAMP-12 also induced β-arrestin recruitment and receptor internalization. Interestingly, C9 inhibited both SP and PAMP-induced β-arrestin recruitment in HTLA cells. C9 also blocked SP and PAMP-12-induced MRGPRX2 internalization but the magnitude of the inhibition was dependent on whether the experiment was performed in transfected RBL-2H3 cells, HTLA cells, or LAD2 cells which endogenously express MRGPRX2. Rocuronium caused robust β-arrestin recruitment, which was inhibited by C9. However, rocuronium induced minimal MRGPRX2 internalization in transfected RBL-2H3 and HTLA cells as well as LAD2 cells. This suggests that depending on the ligand used, β-arrestin recruitment could be dissociated from receptor internalization. Despite this difference, it is clear that C9 inhibits both G protein and β-arrestin-dependent signaling. From a mechanistic standpoint, Cao et al. (25), showed that C9 inhibits basal recruitment of Gαq by MRGPRX2, thus exhibiting inverse agonist activity. Our finding that C9 also causes significant inhibition of constitutive β-arrestin recruitment by the missense MRGPRX2 variant V282M, supports the notion that it serves as an inverse agonist. However, the original compounds (compound 1 and 2) from which C9 was derived inhibit SP binding to MRGPRX2 and block GTP-γS binding activities of a Gα protein (19). Based on these findings, it was proposed that they serve as competitive antagonists of MRPGRX2. Additional studies will be required to determine the exact mechanism *via* which compound 1, 2 and C9 inhibit MRGPRX2-mediated responses in MCs.

In summary, we have shown that C9 is a potent and selective inhibitor of MRGPRX2-mediated MC degranulation using three different assays; cell surface expression of CD63, CD107a and β-hexosaminidase release. MRGPRX2 is highly polymorphic and most of the missense mutants remain uncharacterized (1). We have shown that the V282M missense mutation, which displays a loss of function phenotype for G protein activation and MC degranulation, promotes constitutive activity for β-arrestin recruitment and that C9 inhibits this response. It is possible that certain individuals display a gain of function phenotype for degranulation contributing to MRGPRX2-mediated disorders. It should be feasible to generate MCs from the peripheral blood of these individuals and determine if C9 inhibits constitutive activity. Elevated expression of MRGPRX2 has been reported in skin MCs of patients with allergic contact dermatitis, chronic spontaneous urticaria, rosacea, and mastocytosis (11, 13, 14, 54). Beyond the skin, MRGPRX2-expressing MCs are increased in the gingiva of patients with chronic periodontitis, and lung MCs of individuals who died from asthma (55, 56). Thus, there is tremendous therapeutic potential for utilizing small molecule MRGPRX2 blockers to modulate a variety of MC-mediated disorders. Compound C9 could be the starting point for developing such therapeutics but bioavailability, stability, toxicity, and its possible off-target effects need to be determined. Furthermore, given that C9 does not modulate MrgprB2-mediated MC degranulation, preclinical studies will require humanized mice or transgenic mice that express MRGPRX2 in MCs.

## Data availability statement

The raw data supporting the conclusions of this article will be made available by the authors, without undue reservation.

## Author contributions

HA contributed to the conception and supervision. HA and CO contributed to funding acquisition of the study. MB and AA performed the experiments and analyzed the data. YH and CO provided human skin MCs for the study. MB, AA, and HA wrote the first draft of the manuscript. All authors contributed to the article and approved the submitted version.

## Funding

This work was supported by grants R01-AI143185, R01- AI149487, and R01-AI124182 to HA; and R21-AR067996 and P20 GM-103641 to CO.

## Acknowledgments

We thank the FACS core facility of the University of Pennsylvania School of Dental Medicine for flow cytometry acquisition and analysis.

## Conflict of interest

The authors declare that the research was conducted in the absence of any commercial or financial relationships that could be construed as a potential conflict of interest.

## Publisher’s note

All claims expressed in this article are solely those of the authors and do not necessarily represent those of their affiliated organizations, or those of the publisher, the editors and the reviewers. Any product that may be evaluated in this article, or claim that may be made by its manufacturer, is not guaranteed or endorsed by the publisher.

## Abbreviations

BAT, basophil activation test; FcϵRI, high-affinity immunoglobulin E receptor; FITC, fluorescein isothiocyanate; IgER-CL, IgE receptor cross-linking; GPCR, G Protein-coupled receptor; MCs, mast cells; MFI, mean fluorescent intensity; MrgprB2, Mas-related G protein-coupled receptor B2; MRGPRX2, Mas-related G protein-coupled receptor X2; NMBAs, neuromuscular blocking agents; PAMP-12, pro-adrenomedullin peptide-12; PE, phycoerythrin; PTx, Pertussis toxin; PMCs, peritoneal mast cells; RBL-2H3, rat basophilic leukemia-2H3 cells; RBL-MRGPRX2, RBL-2H3 stably expressing MRGPRX2; rhSCF, recombinant human stem cell factor; MAT, mast cell activation test; MW, molecular weight; SCF, stem cell factor; SP, substance P; TANGO assay, transcriptional activation following arrestin translocation assay; WT, wild-type.
